# Mechanisms linking metabolism of *Helicobacter pylori* to ^18^O and ^13^C-isotopes of human breath CO_2_

**DOI:** 10.1038/srep10936

**Published:** 2015-06-03

**Authors:** Suman Som, Anulekha De, Gourab Dutta Banik, Abhijit Maity, Chiranjit Ghosh, Mithun Pal, Sunil B. Daschakraborty, Sujit Chaudhuri, Subhra Jana, Manik Pradhan

**Affiliations:** 1 Department of Chemical, Biological and Macromolecular Sciences, S. N. Bose National Centre for Basic Sciences, Salt Lake, JD Block, Sector III, Kolkata 700098, India; 2 Department of Gastroenterology, AMRI Hospital, Salt Lake City, JC-16 & 17, Kolkata 700098, India; 3 Department of Gastroenterology, Ruby General Hospital, Kasba Golpark, E. M. Bypass Kolkata, West Bengal 700107, India; 4 Thematic Unit of Excellence on Nanodevice Technology, S. N. Bose National Centre for Basic Sciences, Salt Lake, JD Block, Sector III, Kolkata 700098, India

## Abstract

The gastric pathogen *Helicobacter pylori* utilize glucose during metabolism, but the underlying mechanisms linking to oxygen-18 (^18^O) and carbon-13 (^13^C)-isotopic fractionations of breath CO_2_ during glucose metabolism are poorly understood. Using the excretion dynamics of ^18^O/^16^O and ^13^C/^12^C-isotope ratios of breath CO_2_, we found that individuals with *Helicobacter pylori* infections exhibited significantly higher isotopic enrichments of ^18^O in breath CO_2_ during the 2h-glucose metabolism regardless of the isotopic nature of the substrate, while no significant enrichments of ^18^O in breath CO_2_ were manifested in individuals without the infections. In contrast, the ^13^C-isotopic enrichments of breath CO_2_ were significantly higher in individuals with *Helicobacter pylori* compared to individuals without infections in response to ^13^C-enriched glucose uptake, whereas a distinguishable change of breath ^13^C/^12^C-isotope ratios was also evident when *Helicobacter pylori* utilize natural glucose. Moreover, monitoring the ^18^O and ^13^C-isotopic exchange in breath CO_2_ successfully diagnosed the eradications of *Helicobacter pylori* infections following a standard therapy. Our findings suggest that breath ^12^C^18^O^16^O and ^13^C^16^O^16^O can be used as potential molecular biomarkers to distinctively track the pathogenesis of *Helicobacter pylori* and also for eradication purposes and thus may open new perspectives into the pathogen’s physiology along with isotope-specific non-invasive diagnosis of the infection.

*Helicobacter pylori* (*H. pylori*) is a micro-aerophilic pathogen, which is known to be able to colonize the mucosal surfaces of the human stomach, where it gives rise to chronic gastritis, peptic ulcers^1^,^2^,^3^ and is closely linked to the development of certain types of gastric cancer^4^. The gastric pathogen *H. pylori* uses glucose as the primary energy substrate^5^,^6^, although the overall metabolism of *H. pylori* yet remains inadequately understood. Some early evidences, however, suggest that *H. pylori* has the ability to utilize glucose for metabolism through a glucokinase activity^7^ and enzymes of the pentose phosphate and glycolysis pathways^8^,^9^. Carbon dioxide (CO_2_) is usually produced as a by-product of glucose catabolism which is then transported to the lungs through the blood stream, and finally it is excreted in human breath. However, the precise role of glucose metabolism, especially in the pathogenesis of the *H. pylori* infection is not currently known. A new insight into the role of glucose metabolism is essential to elucidate the pathophysiology of *H. pylori* for its successful colonization of the gastrointestinal tract. However, to our knowledge, so far there have been no studies focused on glucose uptake for individuals harboring *H. pylori* infections, exhibiting the time-dependent excretion dynamics of the metabolite CO_2_ in exhaled breath. The purpose of this study was therefore, primarily to explore the potential links between breath CO_2_ and *H. pylori* infections in response to unlabelled and labelled ^13^C-enriched glucose metabolism. A complete understanding of glucose metabolism during the *H. pylori* infection could be of significance in the development of novel therapies for the micro-organism alongside new and better approaches for treating the most common human bacterial infection.

Furthermore, an earlier study revealed that the oxygen-16 (^16^O) and the oxygen-18 (^18^O) isotopes in ^12^C^16^O_2_ and water (H_2_^18^O), respectively, are rapidly interchanged during the human respiration process mediated by the metalloenzyme carbonic anhydrase (CA)^10^,^11^. It is also known that *H. pylori* encodes two different forms of the metalloenzyme carbonic anhydrase (α-CA and β-CA)^12^ and this gastric pathogen plays a vital role in inter-conversion of carbon dioxide and bicarbonate (CO_2_ + H_2_O↔H^+^ + HCO_3_^−^), catalyzed by the CA activity^12^,^13^,^14^. This efficient activity suggests that investigations of breath ^18^O/^16^O isotopic fractionations of CO_2_ may specifically track the gastric pathogen *H. pylori* and hence may introduce a novel non-invasive strategy in the diagnosis of *H. pylori* infections living in human stomach. As a consequence, we hypothesized that simultaneous monitoring the ^18^O/^16^O and ^13^C/^12^C stable isotope ratios of exhaled breath CO_2_ associated with glucose metabolism in *H. pylori* may act as potential markers for the early detection of *H. pylori* infections or during the preclinical phase of the infections. In view of the fact that *H. pylori* is able to uptake and metabolize glucose confirmed as experimentally^15^ and also by analysing the genome sequence^5^ therefore, there is a pressing need to assess the clinical efficacy of the glucose utilization by *H. pylori* for large-scale screening individuals harboring the micro-organism. In addition, unravelling the precise metabolic pathways involved in causing the isotopic fractionations of ^12^C^16^O^16^O, ^13^C^16^O^16^O and ^12^C^18^O^16^O in human breath during the glucose uptake by *H. pylori* remains a major challenge, whenever an individual is at-risk of developing the disease.

In this study, we first report, the potential links of both ^18^O and ^13^C-stable isotopes of breath CO_2_ with the gastric pathogen *H. pylori* in response to glucose ingestion. We investigated simultaneously the time-dependent excretion dynamics of the ^12^C^18^O^16^O/^12^C^16^O^16^O and ^13^C^16^O^16^O/^12^C^16^O^16^O isotope ratios of breath CO_2_ from individuals with *H. pylori* positive and negative by employing a laser-based integrated cavity output spectroscopy (ICOS) method. We further explored the potential metabolic pathways underlying the glucose utilization in the pathogenesis of *H. pylori* infection and the mechanisms linking breath oxygen-18 and carbon-13 isotopic fractionations of CO_2_ to the gastric pathogen *H. pylori.* Finally, we determined various diagnostic parameters such as optimal diagnostic cut-off values, diagnostic sensitivity and specificity of oxygen-18 and carbon-13 stable isotopes in breath CO_2_ to gain a better insight into the diagnostic efficiency for the non-invasive detection of *H. pylori* infection in real-time.

## Results and Discussion

**Figure 1:**
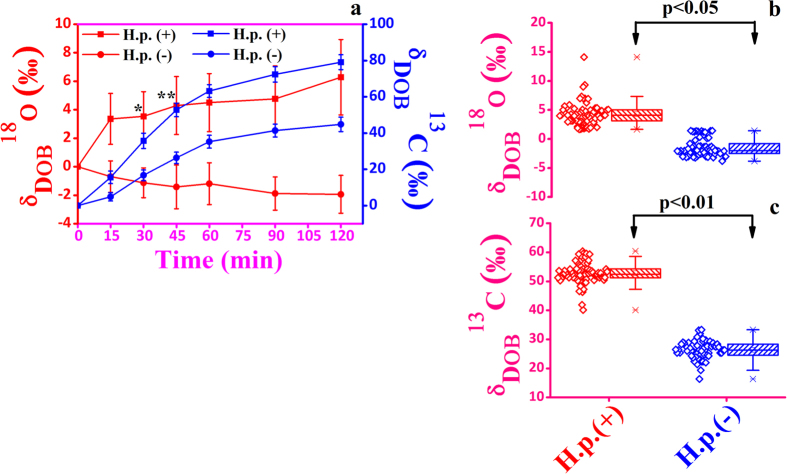
Comparisons of δ_DOB_^18^O‰ and δ_DOB_^13^C‰ values of exhaled breath CO_2_ associated with ^13^C-labelled glucose metabolism in presence [H. p. (+)] and absence [H. p. (−)] of *H. pylori* infection. (**a**) Excretion kinetics of δ_DOB_^18^O‰ and δ_DOB_^13^C‰ values for *H. pylori* positive [H. p. (+)] and *H. pylori* negative [H. p. (−)] individuals up to 120 minutes. (**b**,**c**) The Box-Whiskers plots demonstrating a statistically significant differences of δ_DOB_^18^O‰ [p < 0.05] and δ_DOB_^13^C‰ [p < 0.01] values at 45 minutes between *H. pylori* positive [H. p. (+)] and *H. pylori* negative [H. p. (−)] individuals. *p < 0.05 and **p < 0.01. Data are means ± SE.

To investigate the ^18^O and ^13^C isotopic fractionations of breath CO_2_, we first studied the time-dependent excretion dynamics of both isotopes in exhaled breath after ingestion of an oral dose of ^13^C-enriched glucose for *H. pylori* positive (n = 72) and negative (n = 55) individuals, using a laser-based high-precision cavity-enhanced integrated cavity output spectrometer (ICOS). We explored the isotopic fractionation of CO_2_ by simultaneous monitoring the ^18^O/^16^O and ^13^C/^12^C stable isotope ratios in breath, expressed as delta-over-baseline (DOB) relative to the Vienna Pee Dee Belemnite standard, i.e., δ_DOB_^18^O‰ = [(δ^18^O‰)_t=t_ – (δ^18^O‰)_t=basal_] and δ_DOB_^13^C‰ = [(δ^13^C‰)_t=t_ – (δ^13^C‰)_t=basal_], respectively, associated with glucose metabolism. In this investigation (Fig. 1a), individuals with *H. pylori* positive exhibited significantly higher isotopic enrichments of ^18^O in CO_2_ compared with *H. pylori* negative during the 2h-glucose metabolism, while no significant enrichments of ^18^O in CO_2_ were manifested in individuals without *H. pylori* infections. These findings suggest a potential link between *H. pylori* infections in human stomach and the ^18^O-isotopic fractionations in exhaled breath and hence may open a new route for the non-invasive assessment of *H. pylori* infections. Carbonic anhydrase (CA) activity of *H. pylori* has previously been proposed to interchange the oxygen isotopes of CO_2_ (^16^O) and H_2_O (^18^O) efficiently^10^,^11^, suggesting that in our observations CA activity may play an important role in oxygen-isotope fractionations of breath CO_2_ in the glucose-mediated bacterial environment. Hence a statistically significant difference in the δ_DOB_^18^O‰ values in excretion dynamics established a marked distinction (Fig. 1b) between *H. pylori* infected and non-infected individuals. Taken together, these findings indicate that the monitoring of ^18^O-exchange between C^16^O_2_ and H_2_^18^O in response to CA activity may distinctively track the development of the gastric pathogen in human stomach and might be considered as a potential biomarker for the non-invasive detection of *H. pylori* infection.

We then critically assessed the excretion dynamics of δ_DOB_^13^C (‰) (Fig. 1a) values in exhaled breath samples in response to ^13^C-enriched glucose ingestion. The isotopic enrichments of ^13^C in breath CO_2_ were significantly higher (Fig. 1c) in individuals with *H. pylori* positive compared with individuals with *H. pylori* negative. It was noticed that for *H. pylori* positive patients the δ_DOB_^13^C (‰) values increased gradually with time at a faster rate in comparison with individuals without *H. pylori* infections. Several lines of evidence suggest that *H. pylori* can metabolize glucose in both oxidative and fermentative pathways^8^,^9^ and as a consequence the catabolism of glucose resulted in higher isotopic enrichments of ^13^C in exhaled breath CO_2_. Moreover, in some early evidences^9^,^16^, it was demonstrated the biphasic characteristics of glucose utilization by *H. pylori* with a slower initial period, followed by a second phase with a higher rate of glucose uptake. The transport and utilization of glucose was previously investigated into the intact micro-organism employing the radioactive tracer analysis. Therefore, the gradual increase of the δ_DOB_^13^C (‰) values in the time-dependent excretion dynamics is possibly attributed to the increased rate of glucose utilization through the biphasic activity of the micro-organism. Hence our results of the δ_DOB_^13^C (‰) values in exhaled breath are coincidence with the previous study^16^, where the uptake of glucose into *H. pylori* cells exhibited the biphasic patterns. Our observations therefore, point to new perspectives into the physiology of *H. pylori* underlying the isotopic fractionations of ^13^C in breath CO_2_ associated with glucose metabolism.

**Figure 2:**
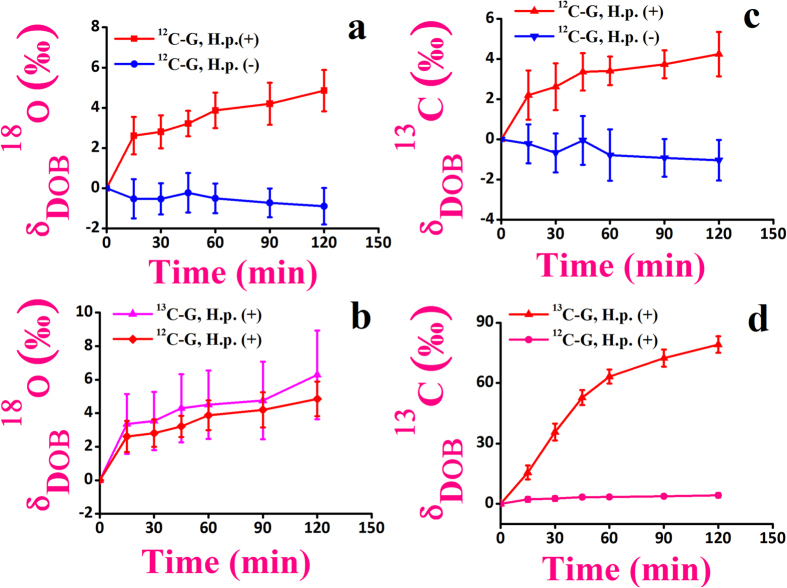
Excretion kinetics of δ_DOB_^18^O‰ and δ_DOB_^13^C‰ values of exhaled breath CO_2_ during unlabelled glucose (^12^C-G) metabolism of *H. pylori* positive [H. p. (+)] and *H. pylori* negative [H. p. (−)] individuals. (**a**) The excretion kinetics illustrating the increased δ_DOB_^18^O‰ values for *H. pylori* positive [H. p. (+)] patients. (**b**) depicts the similar excretion kinetics of δ_DOB_^18^O‰ values with that of ^13^C-enriched glucose. (**c**,**d**) enhancement of δ_DOB_^13^C‰ values for *H. pylori* positive [H. p. (+)] patients and comparisons with the ^13^C-enriched glucose. Values are means ± SE.

We next explored how the time-dependent excretion dynamics of isotopic breath CO_2_ changes after administration of unlabelled glucose (i.e. with no ^13^C-enriched glucose), as the potential role of glucose metabolism in response to unlabelled glucose ingestion for individuals with *H. pylori* infection and the possible links underlying the ^18^O and ^13^C-isotopic fractionations of breath CO_2_ remains unknown. To investigate this, we performed the 2-h excretion kinetics of δ_DOB_^18^O‰ and δ_DOB_^13^C‰ values simultaneously in breath samples for a number of 52 *H. pylori* infected and 45 non-infected individuals. When the unlabelled glucose was orally administered in positive *H. pylori* patients, the post-dose δ_DOB_^18^O‰ values in breath samples manifested a significant change with time and depicted the similar excretion kinetics with that of ^13^C-enriched glucose (Fig. 2a,b), whereas no significant change of the post-dose δ_DOB_^18^O‰ values in breath samples over time was evident for *H. pylori* negative individuals (Fig. 2a). These findings suggest that the mechanisms i.e. oxidation of glucose in the bacterial environment to form bicarbonate (HCO_3_^−^) and subsequently the enzyme carbonic anhydrase-mediated rapid inter-conversion of HCO_3_^−^ and CO_2_, leading to the generation of ^12^C^16^O^18^O, exclusively depends on the substrate (glucose) regardless of its isotopic nature. Interestingly, although the isotopic nature of the substrate is vital to observe effectively the ^13^C-isotopic enrichments of breath CO_2_ (i.e. enhancement of δ_DOB_^13^C‰ values), yet the enrichments of δ_DOB_^13^C‰ values, due to natural abundances of ^13^C, during glucose metabolism of *H. pylori* are significantly distinguishable for *H. pylori* positive patients (Fig. 2c,d), suggesting a new step towards characterizing the transport and utilization of unlabelled glucose into the human pathogen for better understanding of its physiology in the gastric niche. In view of the breath results, we have also established the previous hypothesis^12^ that the bacterium requires high CO_2_ for growth and the interconversion of ^18^O (H_2_^18^O) and ^16^O (C^16^O_2_) is vital, catalyzed by the enzyme carbonic anhydrase activity (α-CA and β-CA) of *H. pylori* and thus this activity might be a contributing factor for the development of the disease in the gastric environment. The summary of the detailed results has been provided in the Table 1.

**Figure 3:**
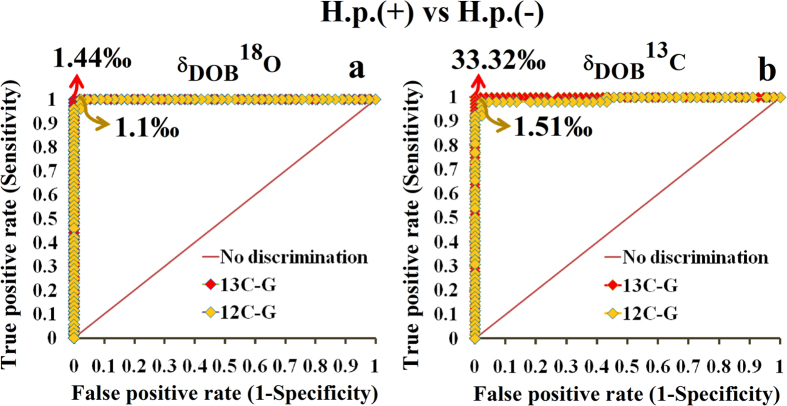
Receiver operating characteristic (ROC) curves analysis for the optimal diagnostic cut-off points of *H. pylori* infection. (**a**) δ_DOB_^18^O values ≥1.44‰ and 1.1‰ are indicative of the *H. pylori* infection associated with ^13^C-labelled and unlabelled glucose metabolism at 45 minute, respectively, whereas (**b**) δ_DOB_^13^C values ≥33.21‰ and 1.51‰ indicate the same for ^13^C-labelled and unlabelled glucose metabolism, respectively.

To distinctively track the *H. pylori* infection as well as for early detection prior to the onset of different gastric diseases, we subsequently determined numerous statistically sound optimal diagnostic cut-off points of δ_DOB_^18^O‰ and δ_DOB_^13^C‰ values in exhaled breath associated with ^13^C-labelled and unlabelled glucose metabolism, using receiver operating characteristics curve (ROC) analysis (Fig. 3). Individuals with δ_DOB_^18^O‰ ≥ 1.44‰ and δ_DOB_^18^O‰ ≥ 1.1‰ were considered to be *H. pylori* positive with and without ^13^C-enriched glucose metabolism respectively, and these corresponded to the diagnostic sensitivity and specificity of 100% and ~98%, respectively. On the contrary, a different optimal diagnostic cut-off point of δ_DOB_^13^C‰ ≥ 33.32‰ between individuals with *H. pylori* positive and negative, demonstrated the sensitivity and specificity of 100% and 100%, respectively, when ^13^C-labelled glucose is ingested, whereas without ^13^C-labelled glucose, δ_DOB_^13^C‰ ≥ 1.51‰ precisely diagnosed the infected and non-infected persons corresponding to the similar levels of diagnostic sensitivity (100%) and specificity (98%). It is noteworthy to mention that the uncertainty of these cut-off values is associated with the less-sensitive techniques for isotope measurements and the variation of isotopic fractionations in the test meal. However, these findings suggest that the oxygen-18 and carbon-13 isotopic fractionations of the major metabolite CO_2_ in human breath linked to glucose metabolism of *H. pylori* provide a new non-invasive approach to treat the world’s most common chronic bacterial infection of humans and hence may have a broad clinical efficacy for precise assessment of the gastric pathogen *H. pylori*.

**Figure 4:**
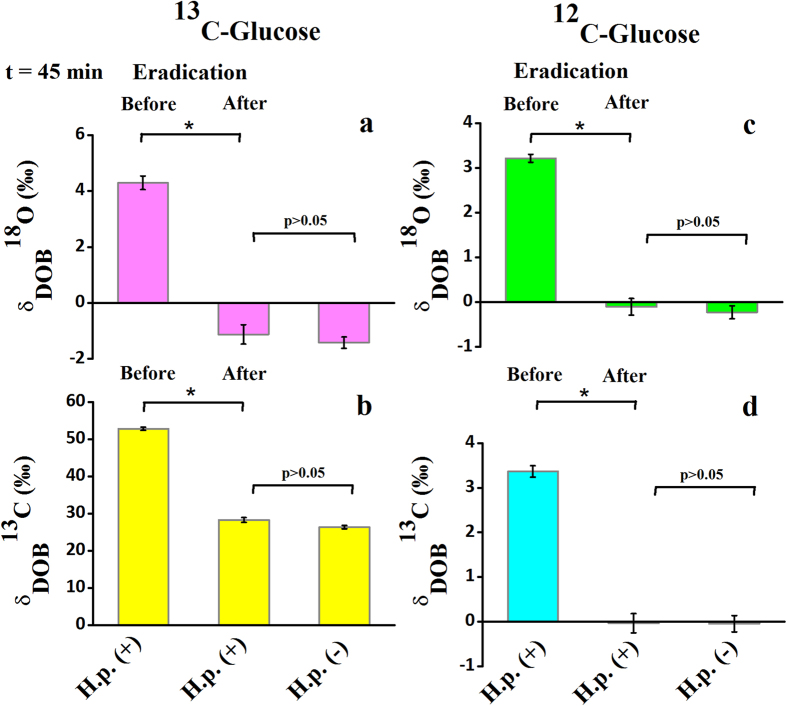
Glucose breath test in response to the standard eradication therapies of the *H. pylori* infection. (**a–d**) a marked distinction (*p < 0.01) for the δ_DOB_^18^O and δ_DOB_^13^C values at 45 minute was observed before and after the therapies in case of both ^13^C-glucose and ^12^C-glucose ingestion.

We next explored the efficacy of the glucose breath test in response to the standard eradication therapies of the infection. A marked depletions of both δ_DOB_^18^O‰ and δ_DOB_^13^C‰ values for *H. pylori* infected patients (n = 37 for ^13^C-glucose and n = 28 for ^12^C-glucose) (Fig. 4) after complete eradication of the infection were manifested, suggesting the widespread clinical significance of the glucose breath test. Our findings associated with the glucose metabolism by *H. pylori* infections thus point towards a considerable clinical advancement in the non-invasive diagnosis of *H. pylori* infection by contrast with the currently available ^13^C-urea breath test (^13^C-UBT), where ^13^C-enriched substrate (urea) is usually used. In view of this result, we therefore posit that the glucose breath test by ingestion of a natural substrate (unlabelled glucose) is a valid and potentially robust new-generation diagnostic tool and thus indicate great promise for comparatively less-expensive and non-toxic global technique, in comparison with the ^13^C-UBT, for the non-invasive assessment i.e. early detection and follow-up of patients after eradication of *H. pylori* infection.

**Figure 5:**
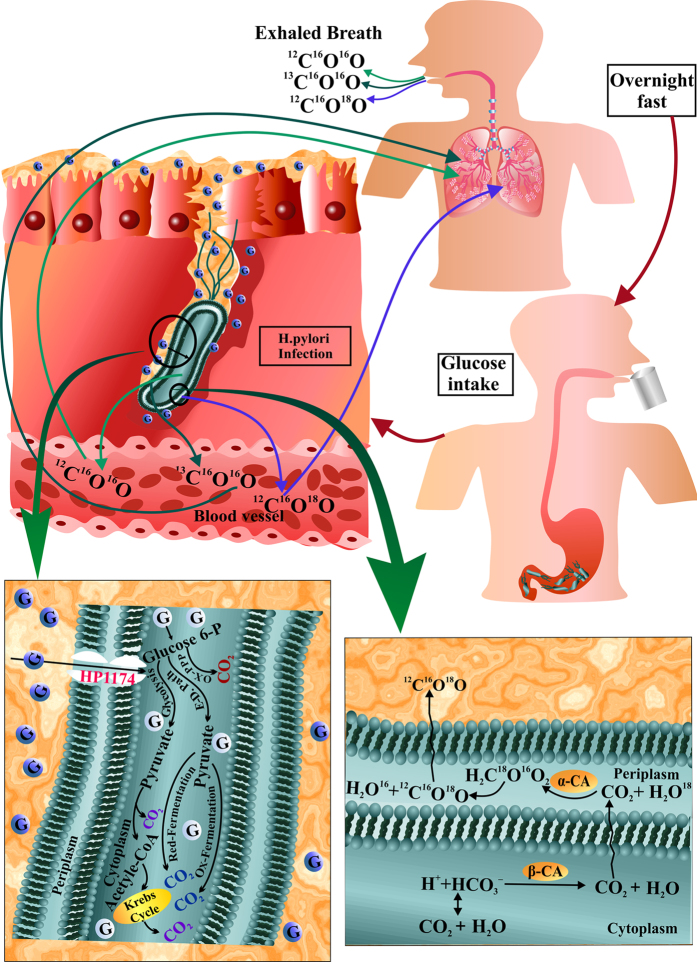
Potential metabolic pathways for ^13^C and ^18^O-isotopic exchanges associated with glucose metabolism by *H. pylori* infection. The ingested glucose in the cytoplasm of *H. pylori* through the HP1174 transporter is phosphorylated to produce glucose-6-phosphate which subsequently incorporated with three potential metabolic routes: glycolysis, pentose phosphate and the Entner-Doudoroff pathway to finally produce major metabolite CO_2_.This major metabolite CO_2_ is then transported through the blood streams and eventually excreted as ^13^C^16^O^16^O and ^12^C^16^O^16^O in exhaled breath. Conversely, the major metabolite CO_2_ diffuses rapidly through the inner member into the periplasm of *H. pylori*, where it forms carbonic acid (H_2_CO_3_), catalyzed by the α-CA. The isotopic exchange between ^16^O of ^12^C^16^O_2_ and ^18^O of H_2_^18^O in response to periplasmic α-CA activity leads to the generation of ^12^C^18^O^16^O, which is then transported to the lungs and is excreted through exhaled breath.

Finally, we elucidated the potential metabolic pathways (Fig. 5) underlying the mechanisms linking isotopic fractionations of breath CO_2_ and glucose utilization by *H. pylori* infection. When a dose of glucose is orally administered to the patients, the ingested glucose disposal takes place in the cytoplasm of *H. pylori* through the HP1174 transporter (protein)^7^. After glucose enters into the cytoplasm, it is phosphorylated to produce glucose-6-phosphate which subsequently incorporated with three potential metabolic routes: glycolysis, pentose phosphate and the Entner-Doudoroff pathway^17^,^18^. A part of the total glucose-6-phosphate, which goes into the pentose phosphate pathway, is predominantly oxidised into CO_2_. The remaining part of glucose-6-phosphate enters into the other two metabolic pathways and may lead to the generation of pyruvate^17^ and eventually gives rise to CO_2_ followed by the formation of acetate as the key metabolite through the intermediary oxidative and reductive fermentation pathways^9^. Another fate for pyruvate is the conversion of acetyl-CoA, which afterwards enters into the Krebs cycle and generates CO_2_ as a by-product^18^. Therefore, the administration of ^13^C-glucose (either from ^13^C-enriched exogenous glucose or naturally abundant ^13^C-glucose) facilitates the production of ^13^CO_2_ in the by-product CO_2_ in presence of *H. pylori* infection. Thereafter, the major metabolite CO_2_ (^13^CO_2_ and ^12^CO_2_) produced by all these metabolic processes is then transported through the blood streams and eventually excreted as ^13^C^16^O^16^O and ^12^C^16^O^16^O in exhaled breath. Conversely, the cytoplasmic β-carbonic anhydrase (β-CA) activity of *H. pylori* catalyzes the reversible interconversion between the major metabolite CO_2_ and HCO_3_^−^ (CO_2_ + H_2_O↔H^+^ + HCO_3_^−^).Then the CO_2_ diffuses rapidly through the inner member into the periplasm of *H. pylori*, where it forms carbonic acid (H_2_CO_3_), catalyzed by the α-CA. Because the isotopes ^16^O of ^12^C^16^O_2_ and ^18^O of H_2_^18^O are rapidly exchanged in response to periplasmic α-CA activity, it therefore leads to the generation of H_2_C^18^O^16^O_2_. This carbonic acid rapidly degasses to produce ^12^C^18^O^16^O, which is then transported to the lungs and is excreted through exhaled breath. As a result, individuals with *H. pylori* infections exhibit the preferential isotopic enrichments of ^18^O in breath CO_2_, whereas no significant change of ^18^O in CO_2_ was manifested in *H. pylori*-uninfected individuals.

In conclusion, our new findings point to a fundamental mechanism underlying both the ^18^O and ^13^C stable isotopic fractionations of the major metabolite CO_2_ in human breath related to glucose metabolism of *H. pylori* infection in humans. Subsequently, we have taken a step towards unravelling the potential metabolic pathways linking the ^18^O and ^13^C-isotopic exchange of breath CO_2_ and the glucose uptake by *H. pylori*, thus suggesting that breath ^12^C^18^O^16^O and ^13^C^16^O^16^O in response to glucose ingestion could be used as potential molecular biomarkers to distinctively track the pathogenesis of *H. pylori* infection in a non-invasive approach. Although many imperative gaps remain in our understanding of these processes and in the pathophysiology underlying the isotopic exchange and glucose metabolism, our studies may provide new perspectives in the isotope-specific molecular diagnosis of *H. pylori* infection and hence may pave the way for broad clinical applications along with eradication purposes following standard therapies. Furthermore, new insight into the mechanism linking the isotopic exchange in breath molecule CO_2_ to glucose metabolism of *H. pylori* is fostering exploration of the molecular basis of this infection and new and better approaches together with new pharmacological targets to prevent or treat the deleterious effects of the world’s most common gastric pathogen.

## Materials and methods

### Subjects

Two hundred and twenty four individuals (135 male and 89 female with average age of 39 ± 10 yrs (SD)) were enrolled for this study with different gastrointestinal disorders such as active peptic ulcer disease (PUD), chronic gastritis, and univestigated dyspepsia. We categorized all the human subjects in two distinct groups: infected with *H. pylori* (*H. pylori* positive patients: 124) and without the infection of *H. pylori* (*H. pylori* negative patients: 100) depending on the reports of gold standard invasive and non-invasive methods, i.e. endoscopy and biopsy based rapid urease test (RUT) and ^13^C-urea breath test (^13^C-UBT).The ^13^C-UBT was considered to be indicative of *H. pylori* positive when δ_DOB_^13^C (‰) ≥ 3‰^19^,^20^,^21^. There were no mismatches between the two test-reports of all the subjects enrolled in this study (Supplementary Table 1). Exclusion criteria included patients with previous history of diabetes and gastric surgery, taking antibiotics, proton pump inhibitors or H_2_ receptor antagonists in the four week prior to endoscopy and ^13^C-UBT. We received the Ethical approval from the Ethics Committee Review Board of AMRI Hospital, Salt Lake, Kolkata, India (Study no.: AMRI/ETHICS/2013/1). The current protocol has also been approved by the institutional administrative of S. N. Bose Centre, Kolkata, India (Ref. no.: SNB/PER-2-6001/13-14/1769) and the methods were carried out in accordance with the approved guidelines. Informed written consents were taken from all patients participating in this study.

### Breath samples collection and measurements

All the human subjects enrolled for the study completed their endoscopic examinations and ^13^C-UBTs, 1-2 days prior to glucose breath test (GBT). On the study day before GBT, all the patients were instructed for their mouth-washing to prevent any kind of contact of ingested test meal with the oral cavity bacteria. After an overnight fasting (10-12 hours), an initial baseline breath sample was collected in a 750 ml breath collection bag (QUINTRON, USA, SL No.QT00892) from each subject. After that a test meal of 75 mg U-^13^C_6_ labelled D-glucose (CIL-CLM-1396-CTM, Cambridge Isotope Laboratories, Inc. USA) or 75 mg unlabeled glucose dissolved in 50 ml water was orally administered to the patient and then subsequent breath samples were collected at 15 minute intervals up to 120 minute. The physical activities of the subjects were restricted inside a room during the test. For the measurements of ^18^O/^16^O and ^13^C/^12^C isotope ratios of exhaled breath CO_2_, a laser-based high-precision ICOS system was employed and the detailed description of the ICOS was given in the following section.

### Integrated cavity output spectrometer (ICOS) for breath analysis

For high precision isotopic measurements of breath CO_2_, a high-resolution carbon dioxide analyzer, based on off-axis integrated cavity output spectroscopy (ICOS) method, has been utilized in this study. The detailed description and the measurement accuracy of ICOS method in comparison to the conventional isotope ratio mass spectrometry (IRMS) have been previously demonstrated elsewhere^22^,^23^. In brief, the laser-based ICOS spectrometer (CCIA 36-EP, Los Gatos research, USA) exploits a high-finesse optical cavity (~59 cm) with two high reflectivity mirrors (R ~ 99.98%) at the both ends of the cavity. This arrangement provides an effective optical path-length of around 3 km through the measuring gas sample, thus offering a high-precision measurement. A continuous wave distributed feedback diode laser operating at ~2.05 μm is repeatedly tuned over 20 GHz to scan the absorption features of ^12^C^16^O^16^O, ^12^C^18^O^16^O and ^13^C^16^O^16^O at the wavenumbers of 4874.448 cm^−1^, 4874.178 cm^−1^ and 4874.086 cm^−1^ respectively. The absorption features of ^12^C^16^O^16^O, ^12^C^18^O^16^O and ^13^C^16^O^16^O, corresponding to the R (27), P (36) and P (16) ro-virational lines respectively, in the (2,0^0^,1) ← (0,0^0^,0) vibrational combinational band of CO_2_, have been utilized to measure the ^13^C/^12^C and ^18^O/^16^O isotope ratios simultaneously. The isotopic enrichments of ^13^CO_2_ and ^12^C^18^O^16^O have been expressed as the conventional notations i.e., δ^13^C (‰) and δ^18^O (‰) respectively, relative to the international standard Pee Dee Belemnite (PDB).
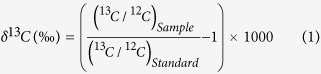

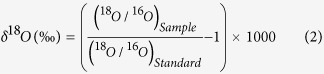
where the standard PDB values for (^13^C/^12^C)_Standard_ and (^18^O/^16^O)_Standard_ are 0.0112372 and 0.0020672, respectively. The accuracy and precision of ICOS method for the δ^13^C‰ measurements were determined by measuring three calibration standards, containing 5% CO_2_ in air analyzed by IRMS (Cambridge Isotope Laboratory, USA), with δ^13^C values ranging from baseline-level (−22.8‰) to high-level (−7.33‰) including the mid-level (−13.22‰) whereas a standard NOAA air tank was used for the calibration of δ^18^O (‰) measurements (Supplementary Table 2 and Supplementary Table 3). A 25 mL breath sample was injected into the ICOS cell with a syringe/stopcock for the measurements. High-purity dry nitrogen (HPNG10-1, F-DGSi SAS, France, purity >99.99%), as the carrier gas, was used to purge the cavity and dilute the breath samples.

### Statistical method

All the data were presented as mean ± SE (Standard Error). For statistical analyses, we performed non-parametric Mann-Whitney test and one way ANNOVA test. A two sided p value <0.05 was taken account as statistically significant of data. Box-Whiskers plots were utilized to demonstrate the statistical distribution of isotopic enrichments of exhaled breath CO_2_. To obtain the optimal diagnostic cut-off values for δ_DOB_^18^O‰ and δ_DOB_^13^C‰ associated with ^13^C-labelled (^13^C-G) and unlabelled glucose (^12^C-G) metabolism, we performed receiver operating characteristic curve (ROC) analysis (Supplementary Table 4, Supplementary Table 5, Supplementary Table 6 and Supplementary Table 7). All the data were analysed using Origin Pro 8.0 (Origin Lab Corporation, USA) and Analyse-it Method Evaluation software (Analyse-it Software Ltd, UK, version 2.30).

## Additional Information

**How to cite this article**: Som, S. *et al.* Mechanisms linking metabolism of *Helicobacter pylori* to ^18^O and ^13^C-isotopes of human breath CO_2_. *Sci. Rep.* **5**, 10936; doi: 10.1038/srep10936 (2015).

## Supplementary Material

Supplementary Information

## Figures and Tables

**Table 1:** The summary of the detailed characteristics of the study subjects for *H. pylori* infected and non-infected groups. UBT and GBT correspond to the urea breath test and glucose breath test respectively.

**Parameters**	**H. pylori infected (mean ± SD)**	**H. pylori non-infected (mean ± SD)**	**p Value**
**AGE**	38.91 ± 10.43	39.19 ± 9.68	0.73
**HbA1c**	5.13 ± 0.12	5.1 ± 0.1	0.07
^**13**^**C-UBT (30 min)**	18.12 ± 12.93	0.67 ± 0.64	<0.001
^**13**^**C-GBT (45 min)**			
δ_DOB_^13^C‰	52.70 ± 3.71	26.34 ± 3.16	<0.001
δ_DOB_^18^O‰	4.29 ± 2.03	−1.41 ± 1.53	<0.01
^**12**^**C-GBT (45 min)**			
δ_DOB_^13^C‰	3.36 ± 0.93	−0.04 ± 1.21	<0.01
δ_DOB_^18^O‰	3.11 ± 0.63	−0.22 ± 0.97	<0.01
