# Supplementary material for: Mechanisms linking metabolism of Helicobacter pylori to 18O and 13C-isotopes of human breath CO2
Source: Sci Rep. 2015 Jun 3;5:10936. doi: 10.1038/srep10936 (PMC4454186; doi:10.1038/srep10936)
Supplement: Supplementary Information [file srep10936-s1.pdf]

Supplementary information for

Mechanisms linking metabolism of *Helicobacter pylori* to ^{18}O and ^{13}C-isotopes of human breath CO_2

Suman Som, Anulekha De, Gourab Dutta Banik, Abhijit Maity, Chiranjit Ghosh, Mithun Pal, Sunil B. Daschakraborty, Sujit Chaudhuri, Subhra Jana, and Manik Pradhan*

*Corresponding Author. Email: manik.pradhan@bose.res.in

Supplementary Table 1

Characteristics of enrolled 224 individuals. The datasheet includes HbA1c (%), Rapid Urease Test (RUT) and ^{13}C-UBT reports of *H. pylori* positive [n=124] and *H. pylori* negative [n=100] individuals. For ^{13}C-UBT results, (+) stands for positive and (-) stands for negative.

**Subjects**	**Sex (M/F)**	**Age (yrs.)**	**HbA1c (%)**	***Endoscopy & Biopsy (Rapid Urease Tests)***	**^{13}C-UBT (with $\delta_{DOB}{}^{13}C$ ‰ at 30 min)**	***Gastrointestinal Disorders***
GP1	F	28	5.1	Positive	5.6 (+)	Active Deodenal Ulcer
GP2	M	42	5.0	Positive	10.45 (+)	Dypepsia
GP3	M	39	5.0	Positive	21.08 (+)	Gastric Ulcer
GP4	F	31	5.0	Positive	18.78 (+)	Dypepsia
GP5	F	24	5.2	Positive	3.95 (+)	Dypepsia
GP6	F	27	5.3	Positive	12.45 (+)	Deodenal Scarring
GP7	F	32	5.1	Positive	17.48 (+)	Active Deodenal Ulcer
GP8	M	48	5.0	Positive	25.12 (+)	Dypepsia
GP9	M	26	5.0	Positive	57.59 (+)	Healing Ulcer

GP10	M	61	5.1	Positive	33.12 (+)	Erosive Gastritis
GP11	M	54	5.0	Positive	7.4 (+)	Dypepsia
GP12	M	42	5.4	Positive	18.78 (+)	Dypepsia
GP13	F	28	5.2	Positive	29.98 (+)	Gastric Ulcer
GP14	F	34	5.1	Positive	41.78 (+)	Active Deodenal Ulcer
GP15	M	56	5.1	Positive	5.45 (+)	Active Deodenal Ulcer
GP16	M	58	5.1	Positive	8.46 (+)	Gastric Ulcer
GP17	M	49	5.1	Positive	14.59 (+)	Dypepsia
GP18	M	53	5.0	Positive	12.73 (+)	Dypepsia
GP19	M	37	5.0	Positive	10.57 (+)	Deodenal Scarring
GP20	F	26	5.1	Positive	6.9 (+)	Peptic Ulcer
GP21	M	45	5.0	Positive	21.87 (+)	Dypepsia
GP22	F	25	5.0	Positive	56.20 (+)	Dypepsia
GP23	M	51	5.0	Positive	4.58 (+)	Deodenal Scarring
GP24	F	35	5.0	Positive	7.11 (+)	Peptic Ulcer
GP25	F	34	5.2	Positive	12.88 (+)	Healing Ulcer
GP26	F	21	5.3	Positive	6.05 (+)	Erosive Gastritis
GP27	M	24	5.4	Positive	18.49 (+)	Dypepsia
GP28	M	57	5.0	Positive	28.77 (+)	Gastric Ulcer
GP29	M	56	5.0	Positive	5.05 (+)	Peptic Ulcer
GP30	M	21	5.1	Positive	4.52 (+)	Dypepsia
GP31	M	29	5.3	Positive	31.44 (+)	Dypepsia
GP32	M	23	5.2	Positive	9.53 (+)	Active Deodenal Ulcer
GP33	M	28	5.1	Positive	12.35 (+)	Peptic Ulcer
GP34	M	32	5.1	Positive	7.24 (+)	Healing Ulcer
GP35	M	57	5.1	Positive	8.4 (+)	Dypepsia
GP36	M	52	5.0	Positive	11.57 (+)	Dypepsia
GP37	M	49	5.0	Positive	21.64 (+)	Dypepsia
GP38	M	38	5.4	Positive	38.22 (+)	Dypepsia
GP39	F	25	5.2	Positive	43.5 (+)	Deodenal Scarring
GP40	F	29	5.0	Positive	16.42 (+)	Peptic Ulcer
GP41	F	42	5.0	Positive	12.09 (+)	Gastric Ulcer
GP42	M	56	5.0	Positive	21.6 (+)	Dypepsia

GP43	F	27	5.0	Positive	8.52 (+)	Dypepsia
GP44	M	48	5.1	Positive	6.02 (+)	Dypepsia
GP45	M	43	5.2	Positive	47.45 (+)	Dypepsia
GP46	M	53	5.2	Positive	32.05 (+)	Gastric Ulcer
GP47	F	30	5.2	Positive	5.49 (+)	Active Deodenal Ulcer
GP48	F	33	5.3	Positive	8.12 (+)	Dypepsia
GP49	M	45	5.3	Positive	4.87 (+)	Active Deodenal Ulcer
GP50	M	29	5.3	Positive	10.2 (+)	Peptic Ulcer
GP51	M	28	5.3	Positive	23.89 (+)	Peptic Ulcer
GP52	F	40	5.3	Positive	15.64 (+)	Gastric Ulcer
GP53	F	28	5.3	Positive	12.7 (+)	Deodenal Scarring
GP54	F	36	5.3	Positive	28.95 (+)	Active Deodenal Ulcer
GP55	F	29	5.3	Positive	16.45 (+)	Deodenitis
GP56	F	37	5.2	Positive	8.13 (+)	Dypepsia
GP57	F	42	5.0	Positive	7.68 (+)	Dypepsia
GP58	F	35	5.1	Positive	16.52 (+)	Dypepsia
GP59	M	50	5.3	Positive	20.1 (+)	Dypepsia
GP60	M	43	5.1	Positive	5.52 (+)	Dypepsia
GP61	M	39	5.0	Positive	8.47 (+)	Active Deodenal Ulcer
GP62	M	27	5.0	Positive	23.38 (+)	Gastric Ulcer
GP63	M	31	5.1	Positive	34.15 (+)	Gastric Ulcer
GP64	M	38	5.4	Positive	8.79 (+)	Peptic Ulcer
GP65	M	36	5.2	Positive	14.28 (+)	Deodenitis
GP66	F	29	5.2	Positive	24.86 (+)	Deodenal Scarring
GP67	F	41	5.0	Positive	8.9 (+)	Active Deodenal Ulcer
GP68	F	37	5.1	Positive	10.76 (+)	Deodenitis
GP69	F	31	5.2	Positive	6.48 (+)	Peptic Ulcer
GP70	F	46	5.0	Positive	7.82 (+)	Active Deodenal Ulcer
GP71	F	33	5.4	Positive	12.76 (+)	Active Deodenal Ulcer
GP72	F	28	5.0	Positive	9.08 (+)	Active Deodenal Ulcer
GP73	F	36	5.1	Positive	48.29 (+)	Peptic Ulcer
GP74	F	31	5.2	Positive	10.08 (+)	Deodenitis
GP75	M	55	5.2	Positive	23.72 (+)	Deodenitis

GP76	M	50	5.1	Positive	13.98 (+)	Dypepsia
GP77	M	54	5.0	Positive	49.05 (+)	Dypepsia
GP78	M	39	5.4	Positive	37.38 (+)	Active Deodenal Ulcer
GP79	F	42	5.2	Positive	15.67 (+)	Peptic Ulcer
GP80	M	56	5.1	Positive	37.08 (+)	Deodenitis
GP81	M	46	5.3	Positive	26.69 (+)	Active Deodenal Ulcer
GP82	M	59	5.2	Positive	8.87 (+)	Dypepsia
GP83	M	46	5.2	Positive	17.9 (+)	Dypepsia
GP84	F	38	5.1	Positive	9.38 (+)	Dypepsia
GP85	F	32	5.2	Positive	10.7 (+)	Dypepsia
GP86	F	48	5.1	Positive	16.71 (+)	Dypepsia
GP87	F	29	5.0	Positive	29.42 (+)	Dypepsia
GP88	F	34	5.1	Positive	39.48 (+)	Dypepsia
GP89	F	28	5.0	Positive	8.97 (+)	Dypepsia
GP90	F	33	5.1	Positive	10.58 (+)	Deodenitis
GP91	M	39	5.2	Positive	37.18 (+)	Active Deodenal Ulcer
GP92	M	43	5.0	Positive	16.29 (+)	Peptic Ulcer
GP93	M	28	5.1	Positive	5.92 (+)	Peptic Ulcer
GP94	M	30	5.0	Positive	16.59 (+)	Peptic Ulcer
GP95	M	55	5.4	Positive	9.47 (+)	Deodenal Scarring
GP96	M	42	5.0	Positive	32.08 (+)	Deodenitis
GP97	F	38	5.1	Positive	56.43 (+)	Active Deodenal Ulcer
GP98	M	49	5.3	Positive	19.27 (+)	Active Deodenal Ulcer
GP99	F	34	5.2	Positive	9.31 (+)	Active Deodenal Ulcer
GP100	M	43	5.0	Positive	17.48 (+)	Active Deodenal Ulcer
GP101	F	46	5.1	Positive	15.4 (+)	Dypepsia
GP102	F	28	5.1	Positive	28.16 (+)	Active Deodenal Ulcer
GP103	M	34	5.2	Positive	33.28 (+)	Gastric Ulcer
GP104	M	51	5.0	Positive	46.5 (+)	Peptic Ulcer
GP105	F	29	5.2	Positive	12.68 (+)	Active Deodenal Ulcer
GP106	F	38	5.3	Positive	8.47 (+)	Active Deodenal Ulcer
GP107	F	36	5.1	Positive	5.05 (+)	Dypepsia
GP108	F	27	5.1	Positive	25.4 (+)	Dypepsia

GP109	M	55	5.2	Positive	18.78 (+)	Deodenitis
GP110	F	43	5.0	Positive	10.45 (+)	Gastric Ulcer
GP111	M	42	5.0	Positive	6.49 (+)	Gastric Ulcer
GP112	F	38	5.0	Positive	4.98 (+)	Dypepsia
GP113	F	26	5.0	Positive	16.73 (+)	Deodenal Scarring
GP114	F	31	5.2	Positive	18.35 (+)	Deodenal Scarring
GP115	M	57	5.2	Positive	9.2 (+)	Deodenitis
GP116	M	33	5.1	Positive	12.78 (+)	Deodenitis
GP117	M	49	5.2	Positive	5.08 (+)	Dypepsia
GP118	M	56	5.3	Positive	8.48 (+)	Peptic Ulcer
GP119	F	32	5.1	Positive	18.45 (+)	Dypepsia
GP120	F	26	5.0	Positive	25.12 (+)	Active Deodenal Ulcer
GP121	F	56	5.1	Positive	7.89 (+)	Peptic Ulcer
GP122	M	30	5.2	Positive	48.18 (+)	Active Deodenal Ulcer
GP123	M	38	5.0	Positive	12.45 (+)	Dypepsia
GP124	M	52	5.0	Positive	4.85 (+)	Dypepsia
GN1	M	46	5.0	Negative	0.28 (-)	X
GN2	M	34	5.2	Negative	0.1 (-)	X
GN3	M	51	5.3	Negative	1.05 (-)	X
GN4	M	49	5.1	Negative	0.89 (-)	X
GN5	F	25	5.0	Negative	1.56 (-)	X
GN6	F	30	5.0	Negative	0.45 (-)	X
GN7	M	43	5.1	Negative	0.09 (-)	X
GN8	F	27	5.2	Negative	1.24 (-)	X
GN9	F	36	5.1	Negative	0.26 (-)	X
GN10	F	29	5.1	Negative	1.25 (-)	X
GN11	M	36	5.1	Negative	1.44 (-)	X
GN12	M	43	5.1	Negative	1.09 (-)	X
GN13	M	51	5.2	Negative	1.21 (-)	X
GN14	F	26	5.0	Negative	0.56 (-)	X
GN15	F	35	5.2	Negative	0.28 (-)	X
GN16	F	33	5.1	Negative	0.73 (-)	X
GN17	M	50	5.0	Negative	0.95 (-)	X

GN18	M	53	5.0	Negative	1.06 (-)	X
GN19	M	49	5.2	Negative	1.46 (-)	X
GN20	F	42	5.3	Negative	0.23 (-)	X
GN21	F	43	5.2	Negative	-0.2 (-)	X
GN22	M	30	5.2	Negative	0.89 (-)	X
GN23	M	29	5.3	Negative	1.68 (-)	X
GN24	F	25	5.4	Negative	1.49 (-)	X
GN25	F	31	5.0	Negative	1.88 (-)	X
GN26	M	28	5.0	Negative	-0.52 (-)	X
GN27	M	42	5.0	Negative	-0.09 (-)	X
GN28	M	58	5.0	Negative	0.88 (-)	X
GN29	F	29	5.1	Negative	-0.47 (-)	X
GN30	M	41	5.2	Negative	1.69 (-)	X
GN31	F	35	5.1	Negative	0.97 (-)	X
GN32	M	48	5.1	Negative	1.58 (-)	X
GN33	F	32	5.2	Negative	0.28 (-)	X
GN34	M	55	5.2	Negative	0.64 (-)	X
GN35	F	28	5.1	Negative	0.76 (-)	X
GN36	F	34	5.2	Negative	0.34 (-)	X
GN37	M	41	5.3	Negative	-0.48 (-)	X
GN38	M	30	5.1	Negative	-0.71 (-)	X
GN39	M	47	5.0	Negative	-0.05 (-)	X
GN40	M	58	5.1	Negative	1.25 (-)	X
GN41	F	27	5.0	Negative	1.66 (-)	X
GN42	F	29	5.0	Negative	1.08 (-)	X
GN43	M	32	5.0	Negative	1.01 (-)	X
GN44	M	55	5.4	Negative	1.25 (-)	X
GN45	F	31	5.2	Negative	1.49 (-)	X
GN46	M	43	5.2	Negative	0.33 (-)	X
GN47	F	28	5.0	Negative	0.86 (-)	X
GN48	M	39	5.3	Negative	0.41 (-)	X
GN49	M	47	5.3	Negative	1.05 (-)	X
GN50	M	58	5.1	Negative	0.55 (-)	X

GN51	M	54	5.0	Negative	-0.3 (-)	X
GN52	F	29	5.0	Negative	0.67 (-)	X
GN53	M	44	5.0	Negative	0.52 (-)	X
GN54	F	31	5.0	Negative	0.43 (-)	X
GN55	M	46	5.1	Negative	1.71 (-)	X
GN56	F	33	5.1	Negative	1.59 (-)	X
GN57	F	28	5.0	Negative	0.88 (-)	X
GN58	M	49	5.1	Negative	-0.19 (-)	X
GN59	M	35	5.0	Negative	0.49 (-)	X
GN60	M	42	5.1	Negative	0.11 (-)	X
GN61	M	46	5.1	Negative	0.46 (-)	X
GN62	M	33	5.1	Negative	1.58 (-)	X
GN63	M	40	5.0	Negative	0.59 (-)	X
GN64	M	52	5.0	Negative	0.98 (-)	X
GN65	F	34	5.0	Negative	1.05 (-)	X
GN66	F	36	5.0	Negative	1.33 (-)	X
GN67	F	26	5.1	Negative	0.92 (-)	X
GN68	M	44	5.1	Negative	-1.07 (-)	X
GN69	M	31	5.2	Negative	0.62 (-)	X
GN70	M	38	5.1	Negative	0.83 (-)	X
GN71	M	51	5.1	Negative	0.24 (-)	X
GN72	M	33	5.1	Negative	0.77 (-)	X
GN73	M	45	5.0	Negative	0.83 (-)	X
GN74	M	30	5.0	Negative	0.49 (-)	X
GN75	M	49	5.0	Negative	0.22 (-)	X
GN76	M	56	5.2	Negative	0.51 (-)	X
GN77	F	35	5.4	Negative	1.05 (-)	X
GN78	M	41	5.0	Negative	-0.07 (-)	X
GN79	M	40	5.1	Negative	-0.58 (-)	X
GN80	M	29	5.2	Negative	0.79 (-)	X
GN81	M	33	5.0	Negative	1.34 (-)	X
GN82	M	60	5.0	Negative	0.92 (-)	X
GN83	M	49	5.0	Negative	1.06 (-)	X

GN84	M	57	5.1	Negative	0.34 (-)	X
GN85	M	39	5.3	Negative	0.21 (-)	X
GN86	M	48	5.0	Negative	-0.03 (-)	X
GN87	M	40	5.0	Negative	1.14 (-)	X
GN88	M	46	5.1	Negative	1.89 (-)	X
GN89	F	35	5.2	Negative	-0.08 (-)	X
GN90	F	24	5.0	Negative	-0.78 (-)	X
GN91	M	41	5.0	Negative	0.36 (-)	X
GN92	M	32	5.1	Negative	0.22 (-)	X
GN93	M	35	5.2	Negative	0.54 (-)	X
GN94	M	26	5.0	Negative	1.28 (-)	X
GN95	M	34	5.1	Negative	1.19 (-)	X
GN96	M	59	5.2	Negative	0.87 (-)	X
GN97	F	27	5.0	Negative	0.29 (-)	X
GN98	M	46	5.0	Negative	-0.15(-)	X
GN99	M	38	5.0	Negative	0.56 (-)	X
GN100	M	29	5.1	Negative	1.09 (-)	X

Supplementary Table 2

Calibration for $\delta^{13}C$‰. Comparisons of the $\delta^{13}C$‰ values measured by ICOS method with the certified calibration standards (Cambridge Isotope Laboratory, USA). SD corresponds to the 1 standard deviation of three successive measurements

Certified $\delta^{13}C$ ‰ calibration standards analyzed by IRMS	$\delta^{13}C$ ‰ ± SD by ICOS
-22.8	-23.0 ± 0.05
-13.22	-13.20 ± 0.05
-7.33	-7.33 ± 0.05

Supplementary Table 3

Measurement Accuracy for $\delta^{18}O$ (‰). Results of $\delta^{18}O$ (‰) measurements of seven flasks filled from a single certified standard NOAA air tank (Serial No.CB10073).

**Flask**	**$\delta^{18}O$ (‰) measured by ICOS method**
Flask-1	-1.02
Flask-2	-0.99
Flask-3	-1.01
Flask-4	-1.00
Flask-5	-1.01
Flask-6	-0.98
Flask-7	-1.00
Avg.	-1.00
NOAA	-1.00
**Standard Deviation**	±0.01

Supplementary Table 4

Receiver operating characteristic curve (ROC) analysis for the optimal diagnostic cut-off value of $\delta_{DOB}{}^{18}O$ (‰) in breath CO_2 associated with ^{13}C-labelled glucose (13C-G) metabolism in presence [H. p. (+)] and absence [H. p. (-)] of *H. pylori* infection. The selected optimal cut-off value has been highlighted. TP : True positive rate, FP : False positive rate, TN : True negative rate, FN : False negative rate.

13C-G [H.p (+) vs H.p (-)]	TP	FP	TN	FN	TP proportion (Sensitivity)	TN proportion (Specificity)
-3.79	70	56	1	0	1.000	0.018
-3.22	70	55	2	0	1.000	0.035
-3.21	70	54	3	0	1.000	0.053
-3.16	70	53	4	0	1.000	0.070
-3.11	70	52	5	0	1.000	0.088
-3.11	70	51	6	0	1.000	0.105
-3.02	70	48	9	0	1.000	0.158
-3.00	70	47	10	0	1.000	0.175
-2.99	70	46	11	0	1.000	0.193
-2.56	70	45	12	0	1.000	0.211
-2.55	70	43	14	0	1.000	0.246
-2.50	70	42	15	0	1.000	0.263
-2.34	70	41	16	0	1.000	0.281
-2.33	70	40	17	0	1.000	0.298
-2.32	70	39	18	0	1.000	0.316
-2.17	70	38	19	0	1.000	0.333
-2.14	70	37	20	0	1.000	0.351
-2.11	70	36	21	0	1.000	0.368
-2.10	70	35	22	0	1.000	0.386
-2.08	70	34	23	0	1.000	0.404
-2.05	70	33	24	0	1.000	0.421
-2.01	70	32	25	0	1.000	0.439
-2.00	70	31	26	0	1.000	0.456
-2.00	70	29	28	0	1.000	0.491
-1.99	70	28	29	0	1.000	0.509
-1.99	70	27	30	0	1.000	0.526
-1.95	70	26	31	0	1.000	0.544
-1.66	70	24	33	0	1.000	0.579
-1.56	70	23	34	0	1.000	0.596
-1.44	70	22	35	0	1.000	0.614
-1.37	70	21	36	0	1.000	0.632

-1.33	70	20	37	0	1.000	0.649
-1.29	70	19	38	0	1.000	0.667
-1.26	70	18	39	0	1.000	0.684
-1.25	70	17	40	0	1.000	0.702
-1.22	70	16	41	0	1.000	0.719
-1.21	70	15	42	0	1.000	0.737
-0.70	70	14	43	0	1.000	0.754
-0.20	70	13	44	0	1.000	0.772
0.11	70	12	45	0	1.000	0.789
0.22	70	11	46	0	1.000	0.807
0.33	70	10	47	0	1.000	0.825
1.19	70	9	48	0	1.000	0.842
1.22	70	8	49	0	1.000	0.860
1.23	70	7	50	0	1.000	0.877
1.28	70	6	51	0	1.000	0.895
1.33	70	5	52	0	1.000	0.912
1.38	70	4	53	0	1.000	0.930
1.38	70	3	54	0	1.000	0.947
1.41	70	2	55	0	1.000	0.965
1.42	70	1	56	0	1.000	0.982
1.44	70	0	57	0	1.000	1.000
1.66	69	0	57	1	0.986	1.000
1.70	68	0	57	2	0.971	1.000
1.74	67	0	57	3	0.957	1.000
1.80	66	0	57	4	0.943	1.000
1.85	65	0	57	5	0.929	1.000
1.89	64	0	57	6	0.914	1.000
1.94	63	0	57	7	0.900	1.000
1.96	62	0	57	8	0.886	1.000
1.99	61	0	57	9	0.871	1.000
2.01	60	0	57	10	0.857	1.000
2.11	59	0	57	11	0.843	1.000
2.42	58	0	57	12	0.829	1.000
2.89	57	0	57	13	0.814	1.000
2.95	55	0	57	15	0.786	1.000
2.99	54	0	57	16	0.771	1.000
3.05	53	0	57	17	0.757	1.000
3.11	52	0	57	18	0.743	1.000
3.11	51	0	57	19	0.729	1.000
3.12	50	0	57	20	0.714	1.000
3.21	49	0	57	21	0.700	1.000
3.21	48	0	57	22	0.686	1.000
3.22	47	0	57	23	0.671	1.000
3.55	46	0	57	24	0.657	1.000
3.55	45	0	57	25	0.643	1.000
3.58	44	0	57	26	0.629	1.000
3.88	43	0	57	27	0.614	1.000
3.99	41	0	57	29	0.586	1.000
3.99	40	0	57	30	0.571	1.000
4.00	39	0	57	31	0.557	1.000

4.00	38	0	57	32	0.543	1.000
4.02	37	0	57	33	0.529	1.000
4.03	36	0	57	34	0.514	1.000
4.10	35	0	57	35	0.500	1.000
4.11	34	0	57	36	0.486	1.000
4.11	33	0	57	37	0.471	1.000
4.12	32	0	57	38	0.457	1.000
4.12	31	0	57	39	0.443	1.000
4.14	30	0	57	40	0.429	1.000
4.15	29	0	57	41	0.414	1.000
4.20	28	0	57	42	0.400	1.000
4.22	27	0	57	43	0.386	1.000
4.25	26	0	57	44	0.371	1.000
4.33	25	0	57	45	0.357	1.000
4.49	24	0	57	46	0.343	1.000
4.55	23	0	57	47	0.329	1.000
4.66	22	0	57	48	0.314	1.000
4.78	21	0	57	49	0.300	1.000
4.88	20	0	57	50	0.286	1.000
4.98	19	0	57	51	0.271	1.000
4.99	18	0	57	52	0.257	1.000
5.01	17	0	57	53	0.243	1.000
5.08	15	0	57	55	0.214	1.000
5.22	14	0	57	56	0.200	1.000
5.31	13	0	57	57	0.186	1.000
5.33	12	0	57	58	0.171	1.000
5.37	11	0	57	59	0.157	1.000
5.66	10	0	57	60	0.143	1.000
5.88	9	0	57	61	0.129	1.000
6.22	8	0	57	62	0.114	1.000
6.94	7	0	57	63	0.100	1.000
6.99	6	0	57	64	0.086	1.000
7.00	4	0	57	66	0.057	1.000
7.33	3	0	57	67	0.043	1.000
8.99	2	0	57	68	0.029	1.000
9.33	1	0	57	69	0.014	1.000
14.12	0	0	57	70	0.000	1.000

Supplementary Table 5

Receiver operating characteristic curve (ROC) analysis for the optimal diagnostic cut-off value of $\delta_{DOB}{}^{18}O$ (‰) in breath CO_2 associated with unlabelled glucose (12C-G) metabolism in presence [H. p. (+)] and absence [H. p. (-)] of *H. pylori* infection. The selected optimal cut-off value has been highlighted. TP : True positive rate, FP : False positive rate, TN : True negative rate, FN : False negative rate.

12C-G [H.p (+) vs.p (-)]	TP	FP	TN	FN	TP proportion (Sensitivity)	TN proportion (Specificity)
-3.51	52	44	1	0	1.000	0.022
-1.95	52	43	2	0	1.000	0.044
-1.51	52	42	3	0	1.000	0.067
-1.45	52	41	4	0	1.000	0.089
-1.37	52	40	5	0	1.000	0.111
-1.33	52	39	6	0	1.000	0.133
-1.22	52	38	7	0	1.000	0.156
-1.01	52	37	8	0	1.000	0.178
-1.00	52	36	9	0	1.000	0.200
-0.88	52	35	10	0	1.000	0.222
-0.74	52	34	11	0	1.000	0.244
-0.67	52	33	12	0	1.000	0.267
-0.66	52	32	13	0	1.000	0.289
-0.63	52	31	14	0	1.000	0.311
-0.55	52	30	15	0	1.000	0.333
-0.54	52	29	16	0	1.000	0.356
-0.54	52	28	17	0	1.000	0.378
-0.53	52	27	18	0	1.000	0.400
-0.48	52	26	19	0	1.000	0.422
-0.45	52	25	20	0	1.000	0.444
-0.44	52	24	21	0	1.000	0.467
-0.41	52	23	22	0	1.000	0.489
-0.40	52	22	23	0	1.000	0.511
-0.22	52	21	24	0	1.000	0.533
-0.21	52	20	25	0	1.000	0.556
-0.11	52	19	26	0	1.000	0.578
-0.10	52	18	27	0	1.000	0.600
0.01	52	17	28	0	1.000	0.622
0.07	52	16	29	0	1.000	0.644
0.21	52	15	30	0	1.000	0.667
0.37	52	14	31	0	1.000	0.689
0.56	52	13	32	0	1.000	0.711

0.63	52	12	33	0	1.000	0.733
0.66	52	11	34	0	1.000	0.756
0.69	52	10	35	0	1.000	0.778
0.70	52	9	36	0	1.000	0.800
0.71	52	8	37	0	1.000	0.822
0.78	52	7	38	0	1.000	0.844
0.86	52	6	39	0	1.000	0.867
0.90	52	5	40	0	1.000	0.889
0.98	52	4	41	0	1.000	0.911
1.06	52	3	42	0	1.000	0.933
1.08	52	2	43	0	1.000	0.956
1.10	52	1	44	0	1.000	0.978
1.18	51	1	44	1	0.981	0.978
1.47	50	1	44	2	0.962	0.978
1.51	50	0	45	2	0.962	1.000
1.76	49	0	45	3	0.942	1.000
2.27	48	0	45	4	0.923	1.000
2.41	47	0	45	5	0.904	1.000
2.41	46	0	45	6	0.885	1.000
2.44	45	0	45	7	0.865	1.000
2.47	44	0	45	8	0.846	1.000
2.53	43	0	45	9	0.827	1.000
2.56	42	0	45	10	0.808	1.000
2.61	41	0	45	11	0.788	1.000
2.76	40	0	45	12	0.769	1.000
2.79	39	0	45	13	0.750	1.000
2.89	38	0	45	14	0.731	1.000
2.93	37	0	45	15	0.712	1.000
2.96	36	0	45	16	0.692	1.000
2.99	35	0	45	17	0.673	1.000
3.00	34	0	45	18	0.654	1.000
3.02	33	0	45	19	0.635	1.000
3.06	32	0	45	20	0.615	1.000
3.10	31	0	45	21	0.596	1.000
3.11	30	0	45	22	0.577	1.000
3.11	29	0	45	23	0.558	1.000
3.12	28	0	45	24	0.538	1.000
3.13	27	0	45	25	0.519	1.000
3.14	26	0	45	26	0.500	1.000
3.15	25	0	45	27	0.481	1.000
3.18	24	0	45	28	0.462	1.000
3.22	22	0	45	30	0.423	1.000
3.25	21	0	45	31	0.404	1.000
3.27	20	0	45	32	0.385	1.000
3.27	19	0	45	33	0.365	1.000
3.33	18	0	45	34	0.346	1.000
3.35	17	0	45	35	0.327	1.000
3.40	16	0	45	36	0.308	1.000
3.44	15	0	45	37	0.288	1.000
3.45	14	0	45	38	0.269	1.000

3.46	13	0	45	39	0.250	1.000
3.54	12	0	45	40	0.231	1.000
3.55	11	0	45	41	0.212	1.000
3.59	10	0	45	42	0.192	1.000
3.62	9	0	45	43	0.173	1.000
3.66	8	0	45	44	0.154	1.000
3.67	7	0	45	45	0.135	1.000
3.68	6	0	45	46	0.115	1.000
3.69	5	0	45	47	0.096	1.000
3.84	4	0	45	48	0.077	1.000
3.88	3	0	45	49	0.058	1.000
3.92	2	0	45	50	0.038	1.000
4.42	1	0	45	51	0.019	1.000
4.55	0	0	45	52	0.000	1.000

Supplementary Table 6

Receiver operating characteristic curve (ROC) analysis for the optimal diagnostic cut-off value of $\delta_{DOB}{}^{13}C$ (‰) in breath CO_2 associated with ^{13}C-labelled glucose metabolism (13C-G) in presence [H. p. (+)] and absence [H. p. (-)] of *H. pylori* infection. The selected optimal cut-off value has been highlighted. TP : True positive rate, FP : False positive rate, TN : True negative rate, FN : False negative rate.

13C-G [H.p (+) vs H.p (-)]	TP	FP	TN	FN	TP proportion (Sensitivity)	TN proportion (Specificity)
16.33	72	54	1	0	1.000	0.018
19.33	72	53	2	0	1.000	0.036
21.36	72	52	3	0	1.000	0.055
21.99	72	51	4	0	1.000	0.073
22.32	72	50	5	0	1.000	0.091
22.32	72	49	6	0	1.000	0.109
22.39	72	48	7	0	1.000	0.127
23.11	72	47	8	0	1.000	0.145
23.20	72	46	9	0	1.000	0.164
23.64	72	45	10	0	1.000	0.182
23.88	72	44	11	0	1.000	0.200
24.25	72	43	12	0	1.000	0.218
24.33	72	42	13	0	1.000	0.236
24.55	72	41	14	0	1.000	0.255
24.69	72	40	15	0	1.000	0.273
25.33	72	39	16	0	1.000	0.291
25.33	72	38	17	0	1.000	0.309
25.33	72	36	19	0	1.000	0.345
25.33	72	35	20	0	1.000	0.364
25.36	72	34	21	0	1.000	0.382
25.96	72	33	22	0	1.000	0.400
25.99	72	32	23	0	1.000	0.418
25.99	72	31	24	0	1.000	0.436
25.99	72	30	25	0	1.000	0.455
26.20	72	29	26	0	1.000	0.473
26.33	72	28	27	0	1.000	0.491
26.33	72	27	28	0	1.000	0.509
26.33	72	26	29	0	1.000	0.527
26.50	72	25	30	0	1.000	0.545
26.99	72	24	31	0	1.000	0.564
27.00	72	23	32	0	1.000	0.582
27.23	72	22	33	0	1.000	0.600

27.33	72	21	34	0	1.000	0.618
27.44	72	20	35	0	1.000	0.636
27.55	72	19	36	0	1.000	0.655
27.65	72	18	37	0	1.000	0.673
27.66	72	17	38	0	1.000	0.691
27.66	72	16	39	0	1.000	0.709
27.84	72	15	40	0	1.000	0.727
28.33	72	13	42	0	1.000	0.764
28.66	72	12	43	0	1.000	0.782
28.77	72	11	44	0	1.000	0.800
28.90	72	10	45	0	1.000	0.818
28.99	72	9	46	0	1.000	0.836
29.32	72	8	47	0	1.000	0.855
29.32	72	7	48	0	1.000	0.873
29.33	72	6	49	0	1.000	0.891
29.74	72	5	50	0	1.000	0.909
30.20	72	4	51	0	1.000	0.927
30.80	72	3	52	0	1.000	0.945
31.97	72	2	53	0	1.000	0.964
33.12	72	1	54	0	1.000	0.982
33.32	72	0	55	0	1.000	1.000
40.09	71	0	55	1	0.986	1.000
41.89	70	0	55	2	0.972	1.000
46.28	69	0	55	3	0.958	1.000
46.55	68	0	55	4	0.944	1.000
47.24	67	0	55	5	0.931	1.000
48.32	66	0	55	6	0.917	1.000
49.25	65	0	55	7	0.903	1.000
49.44	64	0	55	8	0.889	1.000
49.56	63	0	55	9	0.875	1.000
49.64	62	0	55	10	0.861	1.000
49.99	61	0	55	11	0.847	1.000
50.23	60	0	55	12	0.833	1.000
50.24	59	0	55	13	0.819	1.000
50.36	58	0	55	14	0.806	1.000
50.45	57	0	55	15	0.792	1.000
50.55	56	0	55	16	0.778	1.000
50.74	55	0	55	17	0.764	1.000
50.99	54	0	55	18	0.750	1.000
51.13	53	0	55	19	0.736	1.000
51.20	52	0	55	20	0.722	1.000
51.22	51	0	55	21	0.708	1.000
51.23	50	0	55	22	0.694	1.000
51.23	49	0	55	23	0.681	1.000
51.24	48	0	55	24	0.667	1.000
51.25	47	0	55	25	0.653	1.000
51.32	46	0	55	26	0.639	1.000
51.33	45	0	55	27	0.625	1.000
51.36	44	0	55	28	0.611	1.000
51.97	43	0	55	29	0.597	1.000

52.00	42	0	55	30	0.583	1.000
52.10	41	0	55	31	0.569	1.000
52.12	40	0	55	32	0.556	1.000
52.31	39	0	55	33	0.542	1.000
52.34	38	0	55	34	0.528	1.000
52.36	37	0	55	35	0.514	1.000
52.36	36	0	55	36	0.500	1.000
52.37	35	0	55	37	0.486	1.000
52.39	34	0	55	38	0.472	1.000
52.66	33	0	55	39	0.458	1.000
52.82	32	0	55	40	0.444	1.000
52.85	31	0	55	41	0.431	1.000
52.94	30	0	55	42	0.417	1.000
53.00	29	0	55	43	0.403	1.000
53.02	28	0	55	44	0.389	1.000
53.23	27	0	55	45	0.375	1.000
53.33	26	0	55	46	0.361	1.000
53.55	25	0	55	47	0.347	1.000
53.62	24	0	55	48	0.333	1.000
53.65	23	0	55	49	0.319	1.000
53.78	22	0	55	50	0.306	1.000
53.81	21	0	55	51	0.292	1.000
53.87	20	0	55	52	0.278	1.000
54.21	19	0	55	53	0.264	1.000
54.23	18	0	55	54	0.250	1.000
54.25	17	0	55	55	0.236	1.000
54.33	16	0	55	56	0.222	1.000
54.33	15	0	55	57	0.208	1.000
56.21	14	0	55	58	0.194	1.000
56.29	13	0	55	59	0.181	1.000
56.32	12	0	55	60	0.167	1.000
56.77	11	0	55	61	0.153	1.000
56.85	10	0	55	62	0.139	1.000
57.22	9	0	55	63	0.125	1.000
57.23	8	0	55	64	0.111	1.000
57.84	7	0	55	65	0.097	1.000
58.44	6	0	55	66	0.083	1.000
58.57	5	0	55	67	0.069	1.000
59.36	4	0	55	68	0.056	1.000
59.36	3	0	55	69	0.042	1.000
59.50	2	0	55	70	0.028	1.000
59.56	1	0	55	71	0.014	1.000
60.40	0	0	55	72	0.000	1.000

Supplementary Table 7

Receiver operating characteristic curve (ROC) analysis for the optimal diagnostic cut-off value of $\delta_{DOB}{}^{13}C$ (‰) in breath CO_2 associated with unlabelled glucose (12C-G) metabolism in presence [H. p. (+)] and absence [H. p. (-)] of *H. pylori* infection. The selected optimal cut-off value has been highlighted. TP : True positive rate, FP : False positive rate, TN : True negative rate, FN : False negative rate.

12C-G [H.p (+) vs H.p (-)]	TP	FP	TN	FN	TP proportion (Sensitivity)	TN proportion (Specificity)
-2.37	52	43	1	0	1.000	0.023
-2.33	52	42	2	0	1.000	0.045
-2.32	52	40	4	0	1.000	0.091
-1.37	52	39	5	0	1.000	0.114
-1.33	52	38	6	0	1.000	0.136
-1.32	52	37	7	0	1.000	0.159
-1.27	52	36	8	0	1.000	0.182
-1.23	52	35	9	0	1.000	0.205
-1.10	52	34	10	0	1.000	0.227
-1.03	52	33	11	0	1.000	0.250
-1.00	52	32	12	0	1.000	0.273
-0.98	52	31	13	0	1.000	0.295
-0.83	52	30	14	0	1.000	0.318
-0.56	52	29	15	0	1.000	0.341
-0.38	52	28	16	0	1.000	0.364
-0.33	52	27	17	0	1.000	0.386
-0.31	52	26	18	0	1.000	0.409
-0.29	52	25	19	0	1.000	0.432
-0.22	52	24	20	0	1.000	0.455
-0.22	52	23	21	0	1.000	0.477
-0.21	52	22	22	0	1.000	0.500
-0.21	52	21	23	0	1.000	0.523
-0.13	52	20	24	0	1.000	0.545
0.27	52	19	25	0	1.000	0.568
0.31	51	19	25	1	0.981	0.568
0.33	51	18	26	1	0.981	0.591
0.55	51	17	27	1	0.981	0.614
0.81	51	16	28	1	0.981	0.636
0.84	51	15	29	1	0.981	0.659
0.91	51	14	30	1	0.981	0.682
0.92	51	13	31	1	0.981	0.705
0.97	51	12	32	1	0.981	0.727

1.01	51	11	33	1	0.981	0.750
1.03	51	10	34	1	0.981	0.773
1.14	51	9	35	1	0.981	0.795
1.15	51	8	36	1	0.981	0.818
1.22	51	6	38	1	0.981	0.864
1.31	51	5	39	1	0.981	0.886
1.31	51	4	40	1	0.981	0.909
1.47	51	3	41	1	0.981	0.932
1.50	51	2	42	1	0.981	0.955
1.51	51	1	43	1	0.981	0.977
1.83	50	1	43	2	0.962	0.977
1.93	49	1	43	3	0.942	0.977
2.03	48	1	43	4	0.923	0.977
2.09	48	0	44	4	0.923	1.000
2.11	47	0	44	5	0.904	1.000
2.31	46	0	44	6	0.885	1.000
2.33	45	0	44	7	0.865	1.000
2.43	44	0	44	8	0.846	1.000
2.51	43	0	44	9	0.827	1.000
2.66	42	0	44	10	0.808	1.000
2.73	40	0	44	12	0.769	1.000
2.75	38	0	44	14	0.731	1.000
2.81	37	0	44	15	0.712	1.000
2.96	36	0	44	16	0.692	1.000
3.00	35	0	44	17	0.673	1.000
3.06	34	0	44	18	0.654	1.000
3.11	32	0	44	20	0.615	1.000
3.19	31	0	44	21	0.596	1.000
3.22	30	0	44	22	0.577	1.000
3.25	29	0	44	23	0.558	1.000
3.33	28	0	44	24	0.538	1.000
3.34	26	0	44	26	0.500	1.000
3.36	25	0	44	27	0.481	1.000
3.47	24	0	44	28	0.462	1.000
3.48	23	0	44	29	0.442	1.000
3.66	22	0	44	30	0.423	1.000
3.69	21	0	44	31	0.404	1.000
3.71	20	0	44	32	0.385	1.000
3.74	19	0	44	33	0.365	1.000
3.77	18	0	44	34	0.346	1.000
3.78	17	0	44	35	0.327	1.000
3.86	16	0	44	36	0.308	1.000
3.87	14	0	44	38	0.269	1.000
3.88	13	0	44	39	0.250	1.000
3.94	12	0	44	40	0.231	1.000
3.97	11	0	44	41	0.212	1.000
4.01	10	0	44	42	0.192	1.000
4.02	9	0	44	43	0.173	1.000
4.13	8	0	44	44	0.154	1.000
4.22	7	0	44	45	0.135	1.000

4.27	6	0	44	46	0.115	1.000
4.29	5	0	44	47	0.096	1.000
4.33	4	0	44	48	0.077	1.000
4.59	3	0	44	49	0.058	1.000
4.62	2	0	44	50	0.038	1.000
5.11	1	0	44	51	0.019	1.000
5.99	0	0	44	52	0.000	1.000
